# Somatic Mutations in Circulating Cell-Free DNA and Risk for Hepatocellular Carcinoma in Hispanics

**DOI:** 10.3390/ijms22147411

**Published:** 2021-07-10

**Authors:** Jingjing Jiao, Jessica I. Sanchez, Erika J. Thompson, Xizeng Mao, Joseph B. McCormick, Susan P. Fisher-Hoch, P. Andrew Futreal, Jianhua Zhang, Laura Beretta

**Affiliations:** 1 Department of Molecular and Cellular Oncology, The University of Texas MD Anderson Cancer Center, Houston, TX 77030, USA; jingjing099@gmail.com (J.J.); jisanchez@mdanderson.org (J.I.S.); 2 Department of Genetics, The University of Texas MD Anderson Cancer Center, Houston, TX 77030, USA; ejthomps@mdanderson.org; 3 Department of Genomic Medicine, The University of Texas MD Anderson Cancer Center, Houston, TX 77030, USA; xmao1@mdanderson.org (X.M.); afutreal@mdanderson.org (P.A.F.); jzhang22@mdanderson.org (J.Z.); 4 Brownsville Regional Campus, School of Public Health, The University of Texas Health Science Center at Houston, Brownsville, TX 78520, USA; joseph.b.mccormick@uth.tmc.edu (J.B.M.); susan.p.fisher-hoch@uth.tmc.edu (S.P.F.-H.)

**Keywords:** hepatobiliary diseases, disease prevention, health disparities, precision medicine

## Abstract

Hispanics are disproportionally affected by liver fibrosis and hepatocellular carcinoma (HCC). Advanced liver fibrosis is a major risk factor for HCC development. We aimed at identifying somatic mutations in plasma cell-free DNA (cfDNA) of Hispanics with HCC and Hispanics with advanced liver fibrosis but no HCC. Targeted sequencing of over 262 cancer-associated genes identified nonsynonymous mutations in 22 of the 27 HCC patients. Mutations were detected in known HCC-associated genes (e.g., CTNNB1, TP53, NFE2L2, and ARID1A). No difference in cfDNA concentrations was observed between patients with mutations and those without detectable mutations. HCC patients with higher cfDNA concentrations or higher number of mutations had a shorter overall survival (*p* < 0.001 and *p* = 0.045). Nonsynonymous mutations were also identified in 17 of the 51 subjects with advanced liver fibrosis. KMT2C was the most commonly mutated gene. Nine genes were mutated in both subjects with advanced fibrosis and HCC patients. Again, no significant difference in cfDNA concentrations was observed between subjects with mutations and those without detectable mutations. Furthermore, higher cfDNA concentrations and higher number of mutations correlated with a death outcome in subjects with advanced fibrosis. In conclusion, cfDNA features are promising non-invasive markers for HCC risk prediction and overall survival.

## 1. Introduction

The incidence of HCC has nearly tripled in the United States in the past 30 years and continues to rise, faster than for any other cancer in both men and women [1,2,3]. HCC has a dismal prognosis. It has the second lowest 5-year survival (18%), after pancreatic cancer [1]. This is largely due to the fact that the majority of HCC cases are diagnosed at an advanced stage when curative treatment options are limited [3,4]. Indeed, 5-year survival can reach ~70% for early-stage HCC patients who undergo curative therapy [5,6]. Thus, detection of early-stage HCC is a major necessary step in improving overall survival. Over 90% of HCCs develop on a liver with advanced fibrosis, cirrhosis in particular [7], and patients with cirrhosis are the targeted population for screening. Ultrasound and alpha-fetoprotein (AFP), the most broadly used modalities for HCC surveillance in patients with cirrhosis, lack sensitivity and specificity [8]. Novel modalities for HCC early detection are urgently needed, particularly in high-risk populations. Hispanics in South Texas have the highest age-adjusted incidence of HCC in the United States [9]. We also showed that this population has a 4-fold higher prevalence of advanced liver fibrosis and cirrhosis than the general US population, primarily attributable to metabolic disease [10,11]. 

Cell-free DNA (cfDNA), present in plasma or serum samples, is thought to be derived from apoptotic cells as well as cellular secretion [12,13]. More importantly, cfDNA provides a comprehensive view of the tumor genome [14] and has offered unique opportunities as biomarkers for cancer prognosis [15], patient stratification for targeted therapy [16], and treatment monitoring [17,18,19]. Detection of somatic mutations in cfDNA at early tumor stages, including in asymptomatic subjects, have also been reported [20,21,22,23,24]. We reported the detection in cfDNA of *TP53R249S* mutation, a mutation in HCC associated with exposure to aflatoxin [25,26,27,28]. We detected this mutation in cfDNA samples from 5.7% of South Texas Hispanic patients with HCC [29]. We also reported the frequency of *TERT* promoter mutations, the most frequent genetic alteration in HCC [30,31,32], in cfDNA from HCC and cirrhotic patients [33]. Both studies demonstrated that some HCC somatic mutations can be detected in cfDNA of patients with liver cirrhosis, therefore confirming their potential utility in HCC risk assessment and early detection. In this study, we characterized the somatic mutations in cfDNA from South Texas Hispanic patients with HCC and determined whether some of these mutations can be detected in cfDNA from subjects from the South Texas population with advanced liver fibrosis or cirrhosis. 

## 2. Results

### 2.1. Somatic Mutations in cfDNA from Hispanic Patients with HCC

**Figure 1:**
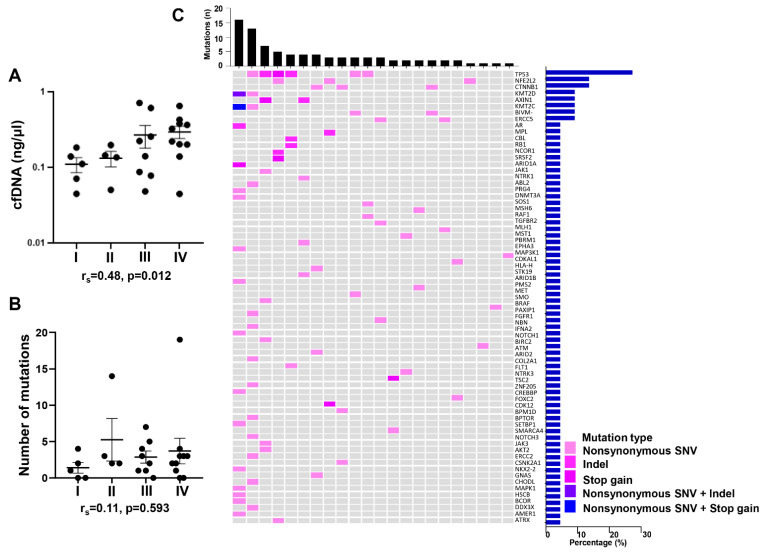
Mutations identified in cfDNA from Hispanic patients with HCC. (**A**) cfDNA concentration in Hispanic HCC patients with different tumor stages. (**B**) Number of mutations detected in cfDNA from Hispanic HCC patients at different tumor stages. (**C**) Genomic landscape of cfDNA from Hispanic subjects with HCC. The heatmap illustrates the nonsynonymous mutations detected in plasma cfDNA. The upper bars represent the number of mutations in each patient. The right bars show the frequencies of the altered genes in HCC subjects with detectable mutations. Data are presented as mean ± SEM. r_s_: spearman correlation coefficient. I: HCC stage 1 (*n* = 5); II: HCC stage 2 (*n* = 4); III: HCC stage 3 (*n* = 8); IV: HCC stage 4 (*n* = 10).

We performed targeted sequencing of over 262 cancer-associated genes using the MD Anderson custom T200.1 platform, on cfDNA from plasma and genomic DNA from buffy coat, collected from 27 Hispanic patients with HCC. The clinical and demographic parameters of these patients are summarized in Supplementary Table S1. The majority of these patients were male (66.7%), with a median age of 68. The prevalence of obesity and diabetes among these patients was high, at 40% and 74.1%, respectively. Approximately a third of these HCCs were in stages I or II. cfDNA concentrations ranged from 0.045 to 0.71 ng/uL, with a median concentration of 0.18 ng/uL. Spearman correlation analysis showed that among all available clinical parameters, cfDNA concentrations only positively correlated with tumor stage (r_s_ = 0.48, *p* = 0.012) (Figure 1A). Indeed, while not statistically significant, cfDNA concentrations tended to increase with tumor stage (Figure 1A). No correlation was observed with tumor burden, age, or AFP concentrations.

Nonsynonymous somatic mutations were identified in 22 out of the 27 patients. While no significant differences in demographic or clinical parameters were observed between the 5 patients without detectable mutations and those 22 patients with detected mutations, patients without detectable mutations had lower AFP values (median: 20.4 vs. 99.6 ng/mL) and were more likely to have histologically differentiated HCCs (Supplementary Table S2. Importantly, no difference in cfDNA concentrations was observed between the two patient groups (Supplementary Table S2). Furthermore, no significant differences in number of mutations detected were observed between tumor stages (Figure 1B). Among the 22 patients with detectable mutations, the number of mutations ranged from 1 to 19, with a median number of 3. The list of all mutations is summarized in Supplementary Table S3. Androgen receptor (AR) p.P12L was the only single mutation found more than once, and both HCCs were at early stage. All detected mutated genes are shown in Figure 1C. Among them, 8 genes were mutated in more than 1 patient. The most commonly mutated gene, detected in 27% of the patients, was TP53, followed by nuclear factor erythroid 2-like 2 (NFE2L2) and beta-catenin 1 (CTNNB1), both detected in 14% of the patients, and then by lysine methyltransferases 2D (KMT2D) and 2C (KMT2C), axin 1 (AXIN1), AR, and BIVM-ERCC5, all detected in 9% of the patients. 

### 2.2. cfDNA Concentrations and Numbers and Clinical Outcome of Hispanic Patients with HCC

**Figure 2:**
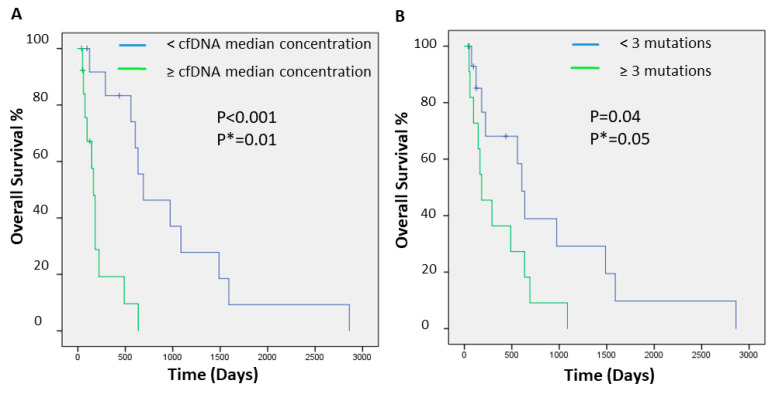
Kaplan–Meier curve of overall survival for HCC patients stratified by cfDNA concentration (**A**) and by number of mutations (**B**). Displayed are the Kaplan–Meier survival curves, and the *p*-values from the log-rank test. *p* *: *p*-value adjusted by tumor stage.

We further evaluated whether concentrations or number of mutations in cfDNA of these patients with HCC could have utility in predicting prognosis. To that end, we calculated and plotted Kaplan–Meier survival curves. Patients with higher concentrations of cfDNA (Figure 2A) or with higher numbers of detected mutations (Figure 2B) had a shorter overall survival (log-rank *p* < 0.001 and *p* = 0.045, respectively). In multivariate analysis adjusting for tumor stage, the association remained significant (log-rank *p*  =  0.010 and 0.057, respectively). 

### 2.3. Somatic Mutations in cfDNA from Hispanic Subjects with Advanced Liver Fibrosis or Cirrhosis 

**Figure 3:**
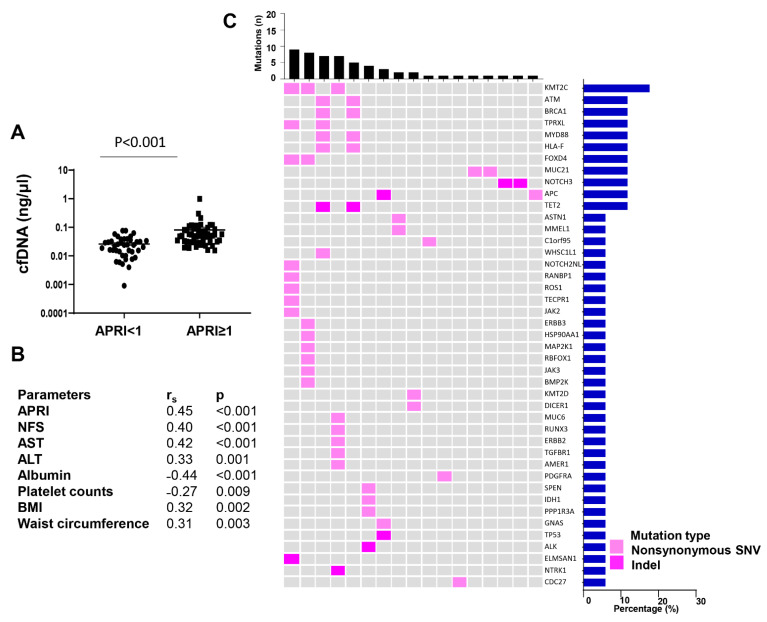
Mutations identified in cfDNA from Hispanic patients with advanced fibrosis/cirrhosis. (**A**) cfDNA concentration in plasma of subjects with APRI < 1 and APRI ≥ 1. (**B**) Clinical parameters of all CCHC subjects. (**C**) Genomic landscape of cfDNA from patients with advanced fibrosis/cirrhosis. Heatmap illustrating nonsynonymous mutations detected in plasma. The upper bar charts represent the number of mutations in each patient. The right bars show the frequencies of specific altered genes in the total cohort. Data are presented as mean ± SEM. APRI, Aspartate transaminase to Platelet Ratio Index; Rs, spearman correlation coefficient; NFS, NAFLD Fibrosis Score; AST, aspartate transaminase; ALT, alanine transaminase; BMI, body mass index.

We also performed targeted sequencing using the same custom T200.1 platform, on cfDNA from plasma and genomic DNA from buffy coat, collected from 51 Hispanic subjects with advanced liver fibrosis or cirrhosis, as determined by aspartate transaminase (AST) to platelet ratio index (APRI) ≥ 1, a non-invasive scoring system for the diagnosis of cirrhosis or advanced liver fibrosis [34,35]. cfDNA and genomic DNA from Hispanic subjects from the same cohort and with APRI < 1 were also extracted and used as controls. The clinical and demographic parameters of these study participants are summarized in Supplementary Table S4. Importantly, there was no significant difference in age and gender between the two groups. Subjects with high APRI were more likely to have abnormal liver function, with higher AST (median: 72.1 vs. 29.5 U/L, *p* < 0.001), higher alanine aminotransferase (ALT) (72.4 vs. 38.3 U/L, *p* < 0.001), and lower albumin (3.8 vs. 4.1 g/dL, *p* < 0.001). HbA1c was also higher in subjects with APRI ≥ 1 than in those with APRI < 1 (median: 6.4% vs. 5.2%, *p* = 0.018), and 49% of those with APRI ≥ 1 were diabetic compared to 28.9% of subjects with APRI < 1. Concentrations of cfDNA were significantly higher in subjects with advanced liver fibrosis/cirrhosis compared to control subjects (median: 0.050 vs. 0.024 ng/uL, *p* < 0.001) (Figure 3A). Furthermore, cfDNA concentrations positively correlated with the non-invasive markers for liver fibrosis, APRI, and NAFLD fibrosis score (NFS) (r_s_ = 0.45 and 0.40, respectively; *p* < 0.001). cfDNA concentrations also positively correlated with AST (r_s_ = 0.42, *p* < 0.001) and ALT (r_s_ = 0.33, *p* = 0.001), and negatively correlated with albumin (r_s_ = −0.44, *p* < 0.001) and platelet counts (r_s_ = −0.27, *p* < 0.009). Interestingly, there was also a positive correlation between cfDNA concentrations and BMI (r_s_ = 0.32, *p* = 0.002), as well as waist circumference (r_s_ = 0.31, *p* = 0.009) (Figure 3B). 

While cfDNA concentrations in control study participants were too low for targeted sequencing, all cfDNAs from subjects with advanced fibrosis/cirrhosis were sequenced successfully. Nonsynonymous mutations were identified in 17 out of the 51 subjects with advanced liver fibrosis/cirrhosis. Among those with detectable mutations, the number of mutations ranged from 1 to 9, with a medium number of 2. Subjects with detectable mutations were more likely to be heavy drinkers (14.3% vs. 8.8%, *p* = 0.029) and less likely to have abnormal ALT (76.5% vs. 94.1%, *p* = 0.009) (Supplementary Table S5). Importantly, no significant differences in cfDNA concentrations nor APRI scores were observed between subjects with detectable mutations and subjects without detectable mutations. The mutated genes are listed in Figure 3C. Among them, 11 genes were detected mutated in more than 1 subject. The list of individual mutations is summarized in Supplementary Table S6. KMT2C was the most commonly mutated gene, occurring in 17.6% of the subjects with detectable mutations, followed by ATM serine/threonine kinase (ATM), BRCA1 DNA repair associated (BRCA1), tetrapeptide repeat homeobox like (TPRXL), MYD88 innate immune signal transduction adaptor (MYD88), major histocompatibility complex class I F (HLA-F), forkhead box D4 (FOXD4), mucin 21, cell surface associated (MUC21), notch receptor 3 (NOTCH3), APC regulator of WNT signaling pathway (APC), and tet methylcytosine dioxygenase 2 (TET2), all occurring in 11.8% of the subjects. Mutations found in more than 1 subject included TET2 p.H860fs, TPRXL p.S219T, MYD88 p.A145T, HLA-F p.Y106C, ATM p.V2079I and p.S2146T, and BRCA1 p.R1347G. 

### 2.4. Concentrations and Number of Mutations in cfDNA and Clinical Outcome of Hispanic Patients with Advanced Liver Fibrosis/Cirrhosis

**Figure 4:**
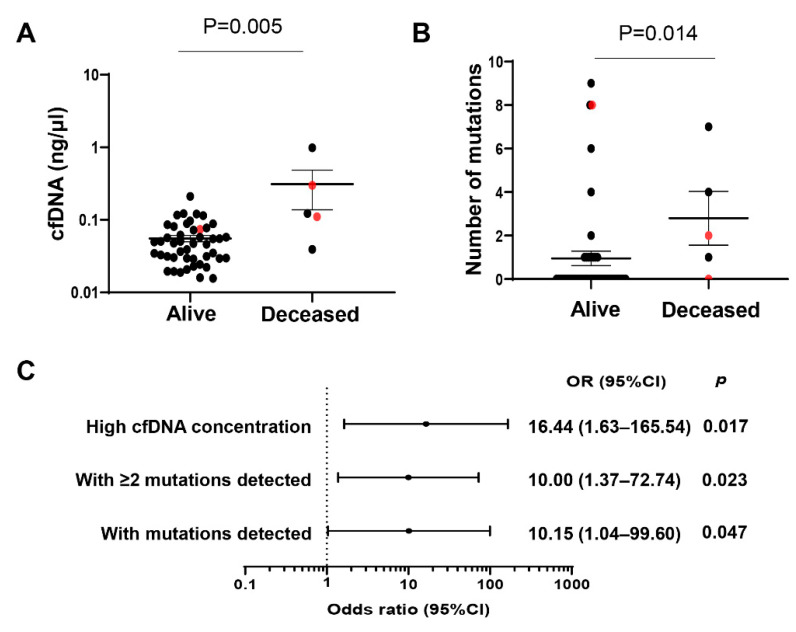
Mutations identified in cfDNA from Hispanic subjects with APRI ≥ 1 correlate with an outcome of death. (**A**) cfDNA concentration in Hispanic subjects with APRI ≥ 1 between alive and deceased. (**B**) Number of mutations detected in cfDNA in Hispanic subjects with APRI ≥ 1 between alive and deceased. Data are presented as mean ± SEM. Subjects with liver cancer are annotated in red. (**C**) Forest plot of significant associations between being deceased and high cfDNA concentration (quartile Q4) and number of mutations among subjects with APRI ≥ 1. APRI, Aspartate transaminase to Platelet Ratio Index; OR, odds ratio; CI, confidence interval.

Among the 51 Hispanic subjects with advanced liver fibrosis/cirrhosis, 5 subjects died: 2 from liver cancer, 1 from colon cancer, and 2 from unknown disease. Subjects who died had significantly higher concentrations of cfDNA than those subjects still currently alive (0.12 vs. 0.048 ng/uL, *p* = 0.005) (Figure 4A). They were also more likely to have detectable mutations in their cfDNA (80% vs. 28.3%, *p* = 0.037) (Figure 4A) and to have a higher number of mutations identified (median: 3 vs. 1, *p* = 0.014) (Figure 4B). In logistic regression analysis, high levels (quartile Q4) of cfDNA concentration were strongly associated with an outcome of death (OR (95% CI): 16.44 (1.63–165.54), *p* = 0.017). Similarly, presence of any mutations (OR (95% CI): 10.15 (1.04–99.60), *p* = 0.047) or at least 2 mutations (OR (95% CI): 10.00 (1.37–72.74), *p* = 0.023) detected in cfDNA was also strongly associated with an outcome of death (Figure 4C).

### 2.5. Comparison of Somatic Mutations in cfDNA between HCC Patients and Subjects with Advanced Liver Fibrosis/Cirrhosis

**Figure 5:**
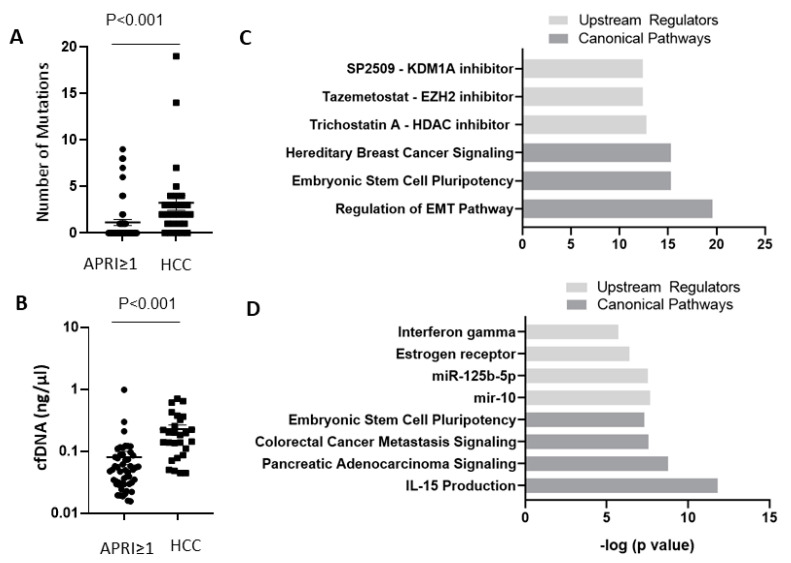
Hispanic patients with HCC have high numbers of mutations (**A**) and higher concentrations of cfDNA (**B**) compared to Hispanic subjects with APRI ≥ 1. Data are presented as mean ± SEM. (**C**,**D**) IPA core analysis identified top canonical pathways and top upstream regulators for mutated genes in cfDNA of Hispanic subjects with HCC (**C**) and mutated genes in cfDNA of Hispanic subjects with APRI ≥ 1 (**D**). APRI, Aspartate transaminase to Platelet Ratio Index.

Overall, mutations were more commonly identified in cfDNA from HCC patients than in cfDNA from subjects with advanced liver fibrosis/cirrhosis (81.5% vs. 33.3%, *p* < 0.001), and when detected, with a higher number of mutations (median: 3 vs. 2, *p* < 0.001) (Figure 5A). In addition, cfDNA concentrations were higher in HCC patients than in subjects with advanced liver fibrosis/cirrhosis (median: 0.18 vs. 0.05 ng/uL, *p* < 0.001) (Figure 5B). A total of 9 genes (TP53, KMT2D, KMT2C, neurotrophic receptor tyrosine kinase 1 (NTRK1), ATM, NOTCH3, Janus Kinase 3 (JAK3), G-protein alpha stimulatory subunit (Gsα subunit) (GNAS), and APC membrane recruitment protein 1 (AMER1)) were found mutated in both cirrhotic/advanced fibrotic subjects and HCC patients. KMT2C p.R894Q was a common mutation identified in both groups. Ingenuity Pathway Analysis (IPA) of all genes found mutated in cfDNA of Hispanic patients with HCC identified the following as predicted upstream regulators: SP2509, an inhibitor of Lysine Demethylase 1A (KDM1A, *p* = 4.05 × 10^−13^), Tazemetostat, an inhibitor of Enhancer Of Zeste 2 Polycomb Repressive Complex 2 Subunit (EZH2, *p* = 4.05 × 10^−13^), and Trichostatin A, an inhibitor of Histone deacetylases (HDAC, *p* = 1.68 × 10^−13^), and as top canonical pathways: hereditary breast cancer signaling (*p* = 5.05 × 10^−16^), embryonic stem cell pluripotency (*p* = 5.04 × 10^−16^), and regulation of EMT pathway (*p* = 2.69 × 10^−20^) (Figure 5C). IPA of all mutated genes identified in cfDNA of Hispanic subjects with advanced liver fibrosis/cirrhosis also identified embryonic stem cell pluripotency (*p* = 4.89 × 10^−8^) as a top canonical pathway. Other top canonical pathways included colorectal cancer metastasis signaling (*p* = 2.74 × 10^−8^), pancreatic adenocarcinoma signaling (*p* = 1.70 × 10^−9^), and IL-15 production (*p* = 1.54 × 10^−12^), while predicted upstream regulators included interferon gamma (*p* = 1.95 × 10^−6^), estrogen receptor (*p* = 4.09 × 10^−7^), miR-125b-5p (*p* = 2.98 × 10^−8^), and mir-10 (*p* = 2.14 × 10^−8^) (Figure 5D).

## 3. Discussion

In this study, we applied targeted next-generation sequencing to the analysis of circulating cfDNA in Hispanics from South Texas with either HCC or advanced fibrosis/cirrhosis. We demonstrated the ability to detect frequently mutated genes in HCC in cfDNA of both HCC patients and cirrhotic/advanced fibrotic subjects. As the disease progressed, cfDNA concentrations and number of mutations increased. In addition, both the number of mutations and the amount of cfDNA correlated with patients’ outcome. Genes found mutated in the cfDNA of Hispanic HCC patients included frequently identified HCC driver mutations, such as TP53, CTNNB1, AXIN1, RB transcriptional corepressor 1 (RB1), AT-rich interaction domain 1A (ARID1A) and 2 (ARID2), SWI/SNF related matrix-associated actin-dependent regulator of chromatin, subfamily A member 4 (SMARCA4), and NFE2L2 [32,36], with TP53 being the most commonly mutated gene. We found that mutations in CTNNB1 were more likely to be identified in patients with alcoholic cirrhosis, which is consistent with the previous reports of association of CTNNB1 mutation with alcoholism [37,38]. A repeated mutation in AR was also found in patients with early-stage HCC and absence of common risk factors, including HCV, HBV, or alcoholic cirrhosis. Although AR mutations are relatively rare events in HCC according to TCGA (occurs in 2.7% of patients), AR has been linked to gender disparity [39] and pathogenesis of HCC in multiple animal models [40,41,42]. Moreover, recently, an AR-driven oncogene, cell cycle-related kinase (CCRK), collaborated with obesity-induced pro-inflammatory signaling to promote NASH-related hepatocarcinogenesis [43]. Given the small number of patients, the identified associations of mutation in cfDNA with HCC risk factors require further validation. 

Major studies of cfDNA for HCC have focused on druggable (actionable) mutations or already known, putative genes with frequent association with HCC [44,45,46,47]. It was reported that cfDNA had a slightly higher efficiency than single tumor specimens (84.2% vs. 78.9%) to identify 19 mutations with therapeutic potential (from NCI-MATCH trial or indicative of target drugs) [45]. cfDNA also has values in predicting treatment efficacy. Targeted sequencing in 13 unresectable HCC cases identified a high percentage (68%) of variants found after sorafenib treatment, indicative of clonal selection [48]. In our study, candidate druggable mutations (e.g., JAK1 and NOTCH3) have also been identified. However, in a study evaluating druggable mutations in 10,000 cancer samples from 62 tumor types, HCC is ranked as second-to-last in terms of prevalence of druggable mutations [49]. 

cfDNA analyses hold promising early diagnostic value for HCC. An assay combining detection of mutations in TP53, CTNNB1, AXIN1, and TERT promoter, HBV integration site, and serum markers AFP and des γ carboxy prothrombin (DCP) was developed. This assay separated HCC from non-HCC with a sensitivity of 85% and a specificity of 93% among individuals who had liver nodules and/or elevated AFP. Furthermore, it could identify early-stage HCC from asymptomatic HBsAg-positive individuals during 6–8 months follow-up [50]. However, this study focused exclusively on Chinese patients with HBV-related HCC. Whether this assay is applicable to other patient populations and etiologies is unknown. Our study provided additional candidate genes which merit further investigation for early diagnosis. Among the mutations identified in the cfDNA of subjects with advanced fibrosis/cirrhosis, somatic mutations in TP53, APC, KMT2C, KMT2D, ATM, and ERBB2 were also reported in the liver with chronic liver diseases, including cirrhosis [51,52,53]. Several genes may play pivotal roles in early stages of hepatocarcinogenesis. Liver-specific disruption of APC leads to activated β-catenin signaling, resulting in hepatomegaly and HCC in mice [54]. NOTCH3 regulates the differentiation of fetal liver stem/progenitor cells into hepatocytes [55] and bile duct development [56]. One subject with advanced fibrosis/cirrhosis had a mutation in the proline–glutamic acid–serine–threonine (PEST) domain (p.P2034fs) of NOTCH3, which leads to activated NOTCH signaling and a subsequent oncogenic event [57]. GNAS is a gene recurrently mutated in inflammatory liver tumors, leading to activation of pSTAT3 [58,59]. 

Besides specific mutations in cfDNA, cfDNA concentrations and number of mutations correlated with disease progression. We also found that cfDNA concentrations and number of mutations correlated with worse outcome in HCC patients, in agreement with a previous report [60]. cfDNA levels and number of mutations increased from advanced fibrosis/cirrhosis to HCC, and cfDNA levels were positively correlated with non-invasive markers for liver fibrosis (APRI and NFS) and degree of hepatic damage (AST, ALT). In the setting of NAFLD, cfDNA levels have been found to correlate with NAFLD severity [61]. In our study, a positive correlation between cfDNA concentrations and obesity or visceral obesity was observed. The results are also consistent with previous publications reporting that cfDNA levels are significantly higher in patients with computed tomography-determined visceral obesity [62], and positively correlated with BMI in HCC patients [63]. 

Stemness and epithelial–mesenchymal transition (EMT) are regarded as two fundamental characteristics of liver cancer stem cells necessary for cancer progression [64]. Remarkably, embryonic stem cell pluripotency was identified by IPA as a top canonical pathways of both genes mutated in cfDNA of HCC subjects and genes mutated in cfDNA in subjects with advanced fibrosis/cirrhosis, suggesting an essential role in both HCC development and HCC progression. It was previously suggested that DNA damage repair signaling is essential for the maintenance of stem cell pluripotency in HCC [65] and that p38 MAPK signaling, leading to F-actin reorganization and activation of nuclear factor erythroid 2-related factor 2-mediated oxidative stress response, collectively contribute to enhanced stemness of HCC cells [66]. EMT was identified as a top canonical pathway only in IPA analysis of HCC mutated genes, confirming its essential role in HCC progression in this study population as well. Interestingly, it was suggested that circulating tumor cells undergoing EMT provide a metric for diagnosis and prognosis of patients with HCC [67]. IPA analysis also suggested a major role of hypoxia, immune surveillance, and natural killer cells through interferon-gamma or IL-15 production, in early events, and highlighted the role of epigenetics in HCC progression. KDM1A forms repressive complexes with HDAC1, regulating differentiation of liver progenitor cells [68,69]. Epigenetic modulation enhances immunotherapy for HCC [70]. EZH2 regulates PD-L1 expression [71] and natural killer cells [72] in HCC.

This study is highly innovative. It is the first description of HCC somatic mutations detected in cfDNA in Hispanics, the population in the United States with the highest incidence of HCC. It is also the first comparative study of somatic mutations detected in cfDNA from subjects with HCC and subjects with advanced liver fibrosis/cirrhosis, including biological pathways associated with the identified mutations. Finally, it is the first report of the potential utility of cfDNA features such as concentration and mutations number in predicting overall survival in non-cancer subjects. 

HCC is a highly heterogenous disease [73,74], with a diversity of low-incidence, nonsynonymous point mutations. In addition to mutations in driver genes, mutations in passenger genes also have the potential to dynamically correlate with tumor burden [75]. The custom panel we used was not specifically designed for HCC known mutated genes. A panel including only driver genes may fail to provide sufficient coverage. However, including more genes in an assay could also greatly increase the cost, a major challenge for cfDNA clinical application for early detection. 

In conclusion, we identified somatic mutations in circulating cfDNA of patients with HCC and in subjects with cirrhosis/advanced fibrosis and therefore at risk for HCC. Together with other cfDNA features such as concentration and mutations number, these mutations could have utility in identifying high-risk subjects for surveillance of HCC.

## 4. Materials and Methods

### 4.1. Study Participants and Sample Collection

The study was approved by the Committee for the Protection of Human Subjects of the University of Texas Health Science Center at Houston and the MD Anderson Cancer Center. Peripheral blood from all study participants was drawn into EDTA tubes. Plasma and separated blood cells were aliquoted and stored at −80 °C until analysis. Biospecimens from 27 Hispanic patients with HCC were obtained from a MD Anderson Cancer Center sample biorepository for patients with histologically confirmed HCC. Demographic and clinical parameters of these patients are shown in Supplementary Table S1. Family history of cancer refers to family history of cancer in first-degree relatives. Fifty-one Hispanics with advanced liver fibrosis or cirrhosis and 41 Hispanics without advanced fibrosis or cirrhosis were selected from the Cameron County Hispanic Cohort (CCHC) [76]. Presence of advanced liver fibrosis/cirrhosis was based on APRI scores ≥ 1. APRI scores were calculated as (AST/33IU/L)/platelet count × 100 [77]. Demographic and clinical parameters of these 92 study participants are shown in Supplementary Table S4. Diabetes was defined using the 2010 American Diabetes Association (ADA) definition [78]. Excess alcohol consumption was defined as >2 drinks/day for men and >1 drink/day for women within 1 year before the completion of data collection. The reported laboratory tests were measured at CLIA-approved reference laboratories. NAFLD fibrosis score (NFS) was calculated as:

NFS = −1.675 + 0.037 × age (year) + 0.094 × BMI (kg/m^2^) + 1.13 × fasting blood glucose ≥ 100/diabetes (yes = 1, no = 0) + 0.99 × AST/ALT ratio − 0.013 × platelet count (×10^9^/L) − 0.66 × albumin (g/dL) (1)

### 4.2. DNA Extraction, Quality Control, and Quantification

Genomic DNA was extracted from 200 µL of buffy coat using the QIAamp DNA blood mini kit (Qiagen Co. Ltd., DE, Düsseldorf, Germany). Circulating cfDNA was extracted from 500 µL of plasma using the QIAamp circulating nucleic acid kit (Qiagen Co. Ltd., DE). DNA quality was assessed using a fragment analyzer and high-sensitivity genomic DNA analysis kit (Advanced Analytical Technologies). cfDNA quantification was performed according to the smear analysis function of the PROSize 2.0 configuration menu (Advanced Analytical Technologies), specifying a size range of 75–225 bp [79]. 

### 4.3. Targeted Sequencing and Bioinformatics Analysis 

Targeted sequencing was performed by the Sequencing and Microarray Facility at MD Anderson Cancer Center. Briefly, indexed libraries were prepared from 500 ng of Diagenode Biorupter sheared genomic DNA (buffy coat) and up to 45 ng of cfDNA (plasma) using the KAPA Hyper Library Preparation Kit (Kapa Biosystems, Inc., Wilmington, MA, USA). Library quality was assessed using the Fragment Analyzer High-Sensitivity NGS Fragment Analysis Kit (Advanced Analytical Technologies). The libraries were then prepared for capture with 8 cycles of pre-LM-PCR amplification. Following pre-LM-PCR, amplified libraries were assessed for quality using the Fragment Analyzer High-Sensitivity NGS Fragment Analysis Kit and quantity using the Qubit dsDNA HS Assay Kit (ThermoFisher, Waltham, MA, USA), then pooled in batches of 3–6 amplified libraries/pool. Targeted capture was performed using a Nimblegen custom targeted solid tumor panel (T200.1) developed at MD Anderson Cancer Center. T200.1 is designed to identify actionable and clinically relevant DNA alteration to cfDNA samples for noninvasive detection of rare mutations in circulating cfDNA. The T200.1 panel covers 262 cancer-associated genes [80]. The enriched libraries were PCR-amplified for 9 cycles post-capture, then assessed for: quality using the Fragment Analyzer, enrichment efficiency by qPCR, and quantified using the Qubit dsDNA HS Assay Kit (ThermoFisher). Sequencing was performed on the HiSeq4000 Sequencer (Illumina Inc., San Diego, CA, USA), 3 samples per lane (6 in 2 lanes), using the 76 nt paired end configuration. The mean coverage of our sample was 322X.

BCL (raw output of Illumina HigSeq) files were processed using Illumina’s Consensus Assessment of Sequence and Variation (CASAVA) tool (Illumina. Available online: http://support.illumina.com/sequencing/sequencing_software/casava.html (accessed on 1 July 2016)) for demultiplexing/conversion to FASTQ format, which is the standard input for most aligners and downstream analytic tools. For DNA samples, the FASTQ files were aligned to the reference genome (human Hg19) using BWA [81] with 3 mismatches, with 2 in the first 40 seed regions for a 76 bases sequencing run. The aligned BAM files were subjected to mark duplication, re-alignment, and re-calibration using Picard and GATK [82] before any downstream analyses. Somatic mutations were found using MuTect [83] and indels using Mutation, and indel results were subjected to filtering by excluding events with less than 20 and 10 bases covering the event for the tumor and normal samples, respectively. For mutations, events with altered base allele frequency less than 2% for cfDNA and greater than 2% in genomic DNA were also excluded. For indels, a 5% tumor allele frequency was used to account for the high false positive rates. Somatic variants were confirmed through visual inspections using IGV (Integrative Genomics Viewer. Available online: http://software.broadinstitute.org/software/igv/ (accessed on 1 September 2016)).

### 4.4. Statistical Analysis

Fisher’s exact tests were used to test the association of mutations with patient characteristics. Overall survival was examined to compare the prognosis of the patients with more than or equal to 3 mutations to those less than 3 mutations. Using SPSS (version 24), the results were displayed as Kaplan–Meier plots with *p*-values from a log-rank test and Cox proportional hazards regression. Logistic regression was performed using SPSS to estimate the odds ratio (OR) and 95% confidence interval (CI) for association of mutations in cfDNA with the worse outcome (death). 

### 4.5. Ingenuity Pathway Analysis

QIAGEN’s Ingenuity^®^ Pathway Analysis (Ingenuity^®^ Pathway Analysis, IPA^®^. Available online: www.qiagen.com/ingenuity (accessed on 17 July 2019)) core analysis was performed using either all genes found mutated in cfDNA of Hispanic patients with HCC or all mutated genes found in cfDNA of Hispanic subjects with cirrhosis/advanced fibrosis. Top upstream regulators and top canonical pathways were graphed using -log(*p*-values) in Graphpad Prism 9.

## Data Availability

The data presented in this study are available upon request from the corresponding author.

## Supplementary Materials

The following are available online at https://www.mdpi.com/article/10.3390/ijms22147411/s1, Table S1: Demographic and clinical parameters of the 27 Hispanic participants with HCC, Table S2: Comparison between Hispanic HCC participants with detected mutations and those without detectable mutations in cfDNA, Table S3: List of somatic mutations detected by targeted sequencing in cfDNA from Hispanic patients with HCC, Table S4: Demographic and clinical parameters of the 51 Hispanic study participants with APRI ≥ 1 and 41 Hispanic study participants with APRI ≤ 1, Table S5: Comparison between the Hispanic subjects with advanced liver fibrosis/cirrhosis with detected mutations and those without detectable mutations, Table S6: List of somatic mutations detected by targeted sequencing in cfDNA in Hispanic study participants with APRI ≥ 1.
