# Supplementary material for: Somatic Mutations in Circulating Cell-Free DNA and Risk for Hepatocellular Carcinoma in Hispanics
Source: Int J Mol Sci. 2021 Jul 10;22(14):7411. doi: 10.3390/ijms22147411 (PMC8304329; doi:10.3390/ijms22147411)
Supplement: Supplementary file 1 [file ijms-22-07411-s001.zip › Supplementary Table S2 Final.pdf]

**Supplementary Table S2. Comparison between Hispanic HCC participants with detected mutations and those without detectable mutations in cfDNA.** Data are presented as frequency (%) or as or as mean (range) - median. BMI: body mass index; AFP: alpha-fetoprotein.

| **Parameters** | **no mutation (n=5)** | **with mutations (n=22)** | **p** |
|---|---|---|---|
| **Male (n=27)** | 4 (80%) | 14 (63.6%) | 0.636 |
| **Age (n=27)** | 66.6 (56.0-73.0) - 69.0 | 66.9 (50.0-88.0) - 66.5 | 0.947 |
| **BMI (n=25)** | 29.8 (24.1-39.3) - 29.0 | 31.6 (23.2-50.7) - 28.5 | 0.663 |
| **Obese (BMI ≥30) (n=25)** | 1 (20%) | 9 (45%) | 0.615 |
| **Diabetes (n=27)** | 3 (60%) | 17 (77.3%) | |
| **Family History of Cancer (n=27)** | 3 (60%) | 10 (45.5%) | 0.648 |
| **Tumor Stage (n=27)** | | | 0.450 |
| **I** | 2 (40%) | 3 (13.6%） | |
| **II** | 0 (0%) | 4 (18.2%) | |
| **III** | 1 (20%) | 7 (31.8%) | |
| **IV** | 2 (40%) | 8 (36.4%) | |
| **Child-Pugh score (n=27)** | | | 0.426 |
| **A** | 3 (60%) | 17 (77.3%) | |
| **B** | 2 (40%) | 5 (22.7%) | |
| **Multiple tumors (n=27)** | 3 (60%) | 12 (54.5%) | >0.999 |
| **AFP (ng/ml) (n=26)** | 630.4 (6.4-2869.0) - 20.4 | 4625.6 (2.8-36000.0) - 99.6 | 0.101 |
| **Differentiation (n=17)** | | | 0.129 |
| **Well** | 3 (100%) | 5 (35.7%) | |
| **Moderate** | 0 (0%) | 5 (35.7%) | |
| **Poor** | 0 (0%) | 4 (28.6%) | |
| **cfDNA concentrations (ng/µl)** | 0.14 (0.11-0.19) - 0.14 | 0.12 (0.04-0.37) - 0.09 | 0.535 |
