# Supplementary material for: Somatic Mutations in Circulating Cell-Free DNA and Risk for Hepatocellular Carcinoma in Hispanics
Source: Int J Mol Sci. 2021 Jul 10;22(14):7411. doi: 10.3390/ijms22147411 (PMC8304329; doi:10.3390/ijms22147411)
Supplement: Supplementary file 1 [file ijms-22-07411-s001.zip › Supplementary Table S3 Final.pdf]

Supplementary Table S3. List of somatic mutations detected by targeted sequencing in cfDNA from Hispanic patients with HCC. Ref_allele: base in the reference genome; Alt_allele: base altered in the sample; Chr.: chromosome; Exonic func: Function of exonic mutation; SNV: single nucleotide variants; Aaannotation: amino acid change; Tumor_f: allele frequency; ns-SNV: nonsynonymous SNV; f-deletion: frameshift deletion; nf-deletion: nonframeshift deletion; f-insertion: frameshift insertion; N: number of subjects with the mutated gene.

Gene (N)	Start_ position	Ref_ allele	Alt_ allele	Chr.	Exonic func	Aaannotation	Tumor_f
**TP53 (6)**	7573982	C	A	17	stopgain	p.E349X	0.35
	7577121	G	A	17	ns-SNV	p.R273C	0.11
	7577534	C	A	17	ns-SNV	p.R249S	0.31
	7578455	C	G	17	ns-SNV	p.A159P	0.27
	7579525	TGA	T	17	f-deletion	p.F69fs	0.38
	7577525	0	-	17	nf-deletion	p.252_252del	0.31
**NFE2L2 (3)**	178097188	C	T	2	ns-SNV	p.D176N	0.05
	178098800	T	A	2	ns-SNV	p.E82V	0.19
	178098975	A	G	2	ns-SNV	p.W24R	0.24
**CTNNB1 (3)**	41266100	T	G	3	ns-SNV	p.R55S	0.15
	41266113	C	G	3	ns-SNV	p.R51T	0.29
	41266124	A	G	3	ns-SNV	p.T41A	0.05
**KMT2D (2)**	49427960	GCA	G	12	f-deletion	p.C3543fs	0.11
	49420661	G	A	12	ns-SNV	p.R5030C	0.05
	49435116	G	A	12	ns-SNV	p.P2146L	0.04
**AXIN1 (2)**	396711	-	A	16	f-insertion	p.F105fs	0.15
	354387	C	A	16	stopgain	p.E391X	0.72
**KMT2C (2)**	151873992	A	G	7	ns-SNV	p.V2849A	0.09
	151921210	C	T	7	stopgain	p.W1071X	0.10
	151932990	C	T	7	ns-SNV	p.R894Q	0.03
**BIVM-ERCC5 (2)**	103515036	G	T	13	ns-SNV	p.A967S	0.30
	103519081	A	G	13	ns-SNV	p.I1261V	0.05
**AR (2)**	66765023	C	T	X	ns-SNV	p.P12L	0.05
							0.10
**MPL (1)**	43817971	GC	G	1	f-deletion	p.S550fs	0.16
**CBL (1)**	119149356	TATG	T	11	nf-deletion	p.455_456del	0.08
**RB1 (1)**	48939048	0	-	13	f-deletion	p.K294fs	0.14
**NCOR1 (1)**	15978938	0	-	17	f-deletion	p.D1210fs	0.45
**SRSF2 (1)**	74732933	0	-	17	f-deletion	p.R102fs	0.26
**ARID1A (1)**	27100375	C	T	1	stopgain	p.Q1363X	0.39
**JAK1 (1)**	65335121	G	A	1	stopgain	p.R174X	0.07
**NTRK1 (1)**	156830772	G	T	1	ns-SNV	p.A16S	0.31
**ABL2 (1)**	179076921	C	T	1	ns-SNV	p.G1161R	0.06
**PRG4 (1)**	186276937	C	A	1	ns-SNV	p.P696T	0.03
**DNMT3A (1)**	25463247	C	T	2	ns-SNV	p.R749H	0.11
**SOS1 (1)**	39249720	T	C	2	ns-SNV	p.M617V	0.08
**MSH6 (1)**	48026327	T	C	2	ns-SNV	p.F402S	0.07
**RAF1 (1)**	12641730	C	T	3	ns-SNV	p.G304D	0.05

**TGFBR2 (1)**	30713231	T	A	3	ns-SNV	p.F211I	0.11
**MLH1 (1)**	37067215	G	T	3	ns-SNV	p.D376Y	0.04
**MST1 (1)**	49724902	C	T	3	ns-SNV	p.R122Q	0.08
**PBRM1 (1)**	52582143	A	G	3	ns-SNV	p.L1669P	0.12
**EPHA3 (1)**	89390941	T	C	3	ns-SNV	p.I336T	0.15
**MAP3K1 (1)**	56155686	C	T	5	ns-SNV	p.R260C	0.04
**CDKAL1 (1)**	21231086	G	T	6	ns-SNV	p.R519I	0.06
**HLA-H (1)**	29856337	A	G	6	ns-SNV	p.D148G	0.06
**STK19 (1)**	31940022	C	A	6	ns-SNV	p.A12E	0.21
**ARID1B (1)**	157150387	G	T	6	ns-SNV	p.M523I	0.06
**PMS2 (1)**	6042115	C	T	7	ns-SNV	p.R169H	0.08
**MET (1)**	116409799	C	T	7	ns-SNV	p.T913M	0.03
**SMO (1)**	128845146	G	T	7	ns-SNV	p.G214C	0.23
**BRAF (1)**	140476824	C	A	7	ns-SNV	p.V528F	0.45
**PAXIP1 (1)**	154760691	G	T	7	ns-SNV	p.A407D	0.11
**FGFR1 (1)**	38279266	G	A	8	ns-SNV	p.T288M	0.07
	38287254	C	T	8	ns-SNV	p.V135I	0.03
**NBN (1)**	90965740	G	T	8	ns-SNV	p.T526K	0.05
**IFNA2 (1)**	21385319	T	C	9	ns-SNV	p.T4A	0.05
**NOTCH1 (1)**	139396752	G	A	9	ns-SNV	p.P1786S	0.05
	139399828	C	T	9	ns-SNV	p.S1507N	0.03
**BIRC2 (1)**	102220619	G	A	11	ns-SNV	p.G12S	0.44
**ATM (1)**	108188108	G	C	11	ns-SNV	p.Q2069H	0.04
**ARID2 (1)**	46245613	C	T	12	ns-SNV	p.P1236L	0.11
**COL2A1 (1)**	48379599	C	A	12	ns-SNV	p.G531V	0.20
**FLT1 (1)**	28897002	C	G	13	ns-SNV	p.V960L	0.05
**NTRK3 (1)**	88680650	T	C	15	ns-SNV	p.N203D	0.07
**TSC2 (1)**	2112534	C	T	16	stopgain	p.Q432X	0.03
**ZNF205 (1)**	3169769	C	T	16	ns-SNV	p.R370W	0.14
**CREBBP (1)**	3779128	G	A	16	ns-SNV	p.R1974W	0.08
**FOXC2 (1)**	86601428	C	G	16	ns-SNV	p.R163G	0.06
**CDK12 (1)**	37687087	C	T	17	stopgain	p.R1331X	0.09
**PPM1D (1)**	58740436	G	T	17	ns-SNV	p.E447D	0.39
**RPTOR (1)**	78899183	C	T	17	ns-SNV	p.A941V	0.05
**SETBP1 (1)**	42531185	G	A	18	ns-SNV	p.R627H	0.07
**SMARCA4(1)**	11134251	C	T	19	ns-SNV	p.R973W	0.03
**NOTCH3 (1)**	15290031	G	A	19	ns-SNV	p.R1175W	0.04
**JAK3 (1)**	17945698	C	T	19	ns-SNV	p.G721D	0.11
**AKT2 (1)**	40745771	C	T	19	ns-SNV	p.G274R	0.34
**ERCC2 (1)**	45864989	C	T	19	ns-SNV	p.A37T	0.06
**CSNK2A1 (1)**	476415	A	G	20	ns-SNV	p.M153T	0.30
**NKX2-2 (1)**	21492791	G	A	20	ns-SNV	p.P198S	0.06
**GNAS (1)**	57429031	C	A	20	ns-SNV	p.S237R	0.18
**CHODL (1)**	19638292	T	A	21	ns-SNV	p.S253R	0.09
**MAPK1 (1)**	22160227	C	T	22	ns-SNV	p.R135K	0.19
**HSCB (1)**	29138132	C	T	22	ns-SNV	p.P17S	0.12
**BCOR (1)**	39916459	T	C	X	ns-SNV	p.H1515R	0.06
**DDX3X (1)**	41203670	T	C	X	ns-SNV	p.F332S	0.12
**AMER1 (1)**	63412518	A	T	X	ns-SNV	p.F217I	0.07
**ATRX (1)**	76907627	G	A	X	ns-SNV	p.R1512C	0.54
