# Supplementary material for: Somatic Mutations in Circulating Cell-Free DNA and Risk for Hepatocellular Carcinoma in Hispanics
Source: Int J Mol Sci. 2021 Jul 10;22(14):7411. doi: 10.3390/ijms22147411 (PMC8304329; doi:10.3390/ijms22147411)
Supplement: Supplementary file 1 [file ijms-22-07411-s001.zip › Supplementary Table S4 Final.pdf]

Supplementary Table S4: Demographic and clinical parameters of the 51 Hispanic study participants with APRI≥1 and 41 Hispanic study participants with APRI<1. BMI: body mass index; HbA1c: hemoglobin A1c; HBV: hepatitis B virus; HCV: hepatitis C virus; AST, aspartate aminotransferase; ALT, alanine aminotransferase; FBG, fasting blood glucose; APRI, aspartate aminotransferase-to-platelet ratio index. Data are presented as mean (range) - median or frequency (%).

**Parameters**	**APRI<1(n=41)**	**APRI≥1(n=51)**	**p**
**APRI (n=92)**	0.4 (0.1-0.9) - 0.4	3.0 (1.0-14.2) - 1.8	<0.001
**Male (n=92)**	17 (41.5%)	22 (43.1%)	0.560
**Age (n=92)**	50.4 (21.0-72.0) - 54.2	51.9 (21.0-83.0) - 52.8	0.691
**BMI (n=92)**	33.9 (22.6-59.4) - 31.4	33.7 (20.3-60.2) - 30.2	0.943
**Obese (BMI ≥30) (n=92)**	21 (51.2%)	31 (60.8%)	0.501
**HbA1c (%) (n=86)**	5.2 (3.5-8.1) - 5.5	6.4 (2.9-14.0) - 5.7	0.018
**Diabetic groups (n=89)**			0.428
**Normal**	12 (31.6%)	7 (13.7%)	
**Prediabetic**	15 (39.5%)	19 (37.3%)	
**Diabetic**	11 (28.9%)	25 (49.0%)	
**Waist circumference (cm) (n=92)**	110.7 (81.0-153.0) - 105.1	112.8 (79.0-151.0) - 105.0	0.735
**NAFLD score (n=79)**	-0.6 (-4.8-3.3) - -0.5	1.3 (-3.0-4.7) - 1.3	0.003
**Drinks per week (n=89)**	3.0 (0.0-44.0) - 0.0	4.7 (0.0-42.8) - 0.0	0.572
**Drinking status (n=89)**			0.550
**Never**	27 (65.9%)	36 (75.0%)	
**Moderate**	12 (29.3%)	7 (14.6%)	
**Heavy**	2 (4.9%)	5 (10.4%)	
**Smoking status (n=91)**			0.317
**Never**	25 (61.0%)	31 (62.0%)	
**Former**	9 (22.0%)	10 (20.0%)	
**Current**	7 (17.1%)	9 (18.0%)	
**Blood tests**			
AST (U/L) (n=92)	30.9 (12.0-69.0) - 29.5	90.9 (18.0-289.0) - 72.1	<0.001
Abnormal AST (>33 U/L) (n=92)	19 (46.3%)	50 (98.0%)	<0.001
ALT (U/L) (n=92)	41.2 (16.0-120.0) - 38.3	97.5 (13.0-366.0) - 72.4	<0.001
Abnormal ALT (>40 U/L male, >31 U/L female) (n=92)	21 (51.2%)	45 (88.2%)	0.008
Albumin (g/dL) (n=82)	4.2 (3.1-4.8) - 4.1	3.7 (2.5-4.5) - 3.8	<0.001
Alkaline phosphatase (U/L) (n=63)	56.9 (5.0-105.0) - 70.3	64.2 (4.0-477.0) - 13.8	0.729
FBG (mg/dL) (n=89)	123.8 (85.0-220.0) - 108.1	147.1 (54.0-359.0) - 118.3	0.299
Insulin (mU/L) (n=92)	25.0 (3.5-71.5) - 16.8	26.1 (4.2-279.0) - 15.4	0.902
Insulin resistance (HOMA) (n=89)	8.2 (1.2-24.7) - 4.6	9.6 (1.2-104.7) - 6.0	0.634
Platelet counts ($\times 10^9$/L) (n=92)	228.2 (161.0-362.0) - 224.4	139.9 (10.0-292.0) - 141.3	<0.001
