# Supplementary material for: Somatic Mutations in Circulating Cell-Free DNA and Risk for Hepatocellular Carcinoma in Hispanics
Source: Int J Mol Sci. 2021 Jul 10;22(14):7411. doi: 10.3390/ijms22147411 (PMC8304329; doi:10.3390/ijms22147411)
Supplement: Supplementary file 1 [file ijms-22-07411-s001.zip › Supplementary Table S1 Final.pdf]

Supplementary Table S1: Demographic and clinical parameters of the 27 Hispanic study participants with HCC. Data are presented as frequency (%) or as mean (range) - median. BMI: body mass index; AFP: alpha-fetoprotein.

**Parameters**	
**Male (n=27)**	18 (66.7%)
**Age (n=27)**	66.9 (50.0-88.0) - 68.0
**BMI (n=25)**	31.2 (23.2-50.7) - 29.0
**Obese (BMI ≥30)**	10 (40%)
**Diabetes (n=27)**	20 (74.1%)
**Family History of Cancer (n=27)**	13 (48.1%)
**Stage (n=27)**	
**I**	5 (18.5%)
**II**	4 (14.8%)
**III**	8 (29.6%)
**IV**	10 (37.0%)
**Child-Pugh Score (n=27)**	
**A**	20 (74.1%)
**B**	7 (25.9%)
**Multiple tumors (n=25)**	15 (55.6%)
**AFP (n=26)**	3857.2 (2.8-36000.0) - 89.8
**Differentiation (n=17)**	
**Well**	8 (47.1%)
**Moderate**	5 (29.4%)
**Poor**	4 (23.5%)
