# Supplementary material for: Somatic Mutations in Circulating Cell-Free DNA and Risk for Hepatocellular Carcinoma in Hispanics
Source: Int J Mol Sci. 2021 Jul 10;22(14):7411. doi: 10.3390/ijms22147411 (PMC8304329; doi:10.3390/ijms22147411)
Supplement: Supplementary file 1 [file ijms-22-07411-s001.zip › Supplementary Table S5 Final.pdf]

Supplementary Table S5. Comparison between the Hispanic subjects with advanced liver fibrosis/cirrhosis with detected mutations and those without detectable mutations. Data are presented as mean (range) - median or frequency (%). BMI: body mass index; HbA1c: hemoglobin A1c; AST: aspartate aminotransferase; ALT: alanine aminotransferase; FBG: fasting blood glucose; NFS: NAFLD fibrosis score

Parameters	no mutation (n=34)	with mutations (n=17)	p
**APRI**	3.6 (1.0-14.2) - 1.6	1.8 (1.0-4.2) - 1.7	0.052
**Male**	12 (35.3%)	10 (58.8%)	0.144
**Age**	54.1 (23-72) - 56.1	46.9 (21-83) - 41.4	0.165
**BMI**	31.6 (24.8-60.2) - 29.4	38.4 (20.3-55.6) - 38.1	0.084
**Obese (BMI ≥30)**	20 (58.8%)	11 (64.7%)	0.075
**HbA1c (%)**	6.4 (3.3-14.0) - 5.8	6.4 (2.9-13.1) - 5.2	0.998
**Diabetic groups**			0.567
Normal	5 (14.7%)	2 (11.8%)	
Prediabetic	11 (32.4%)	8 (47.1%)	
Diabetic	18 (52.9%)	7 (41.2%)	
**Waist circumference (cm)**	109.5 (89.0-142.0) - 102.6	120.2 (79.0-151.0) - 120.6	0.233
**NFS**	1.1 (-3.0-4.6) - 1.2	1.6 (-2.0-4.7) - 1.2	0.568
**Drinks per week**	4.9 (0.0-42.0) - 0.0	3.9 (0.0-42.8) - 0.0	0.748
**Drinking status**			0.029
Never	25 (73.5%)	11 (78.6%)	
Moderate	6 (17.6%)	1 (7.1%)	
Heavy	3 (8.8%)	2 (14.3%)	
**Smoking status**			0.131
Never	20 (58.8%)	11 (68.8%)	
Former	9 (26.5%)	1 (6.3%)	
Current	5 (14.7%)	4 (25.0%)	
**Blood tests**			
AST (U/L)	89.3 (38.0-258.0) - 73.1	94.5 (18.0-289.0) - 57.9	0.834
Abnormal AST (>33 U/L)	34 (100.0%)	16 (94.1%)	NA
ALT (U/L)	90.7 (24.0-366.0) - 72.5	112.5 (13.0-323.0) - 64.8	0.496
Abnormal ALT (>40 U/L male, >31 U/L female)	32 (94.1%)	13 (76.5%)	0.009
Albumin (g/dL)	3.7 (2.5-4.5) - 3.8	3.8 (3.2-4.5) - 3.7	0.775
Alkaline phosphatase (U/L)	56.6 (4.0-207.0) - 16.7	84.7 (4.0-477.0) - 11.3	0.530
FBG (mg/dL)	149.5 (54.0-359.0) - 124.8	141.8 (86.0-327.0) - 116.6	0.792
Insulin (mU/L)	20.1 (4.2-279.0) - 12.8	39.6 (4.6-76.5) - 28.7	0.557
Insulin resistance (HOMA)	7.3 (1.2-104.7) - 3.5	14.8 (1.2-41.7) - 9.1	0.487
Platelet counts ($\times 10^9$/L)	135.5 (10.0-238.0) - 141.6	149.7 (41.0-292.0) - 138.2	0.623
cfDNA concentrations	0.05 (0.02-0.12) - 0.03	0.08 (0.01-0.98) - 0.04	0.423
