# Supplementary material for: Somatic Mutations in Circulating Cell-Free DNA and Risk for Hepatocellular Carcinoma in Hispanics
Source: Int J Mol Sci. 2021 Jul 10;22(14):7411. doi: 10.3390/ijms22147411 (PMC8304329; doi:10.3390/ijms22147411)
Supplement: Supplementary file 1 [file ijms-22-07411-s001.zip › Supplementary Table S6 Final.pdf]

Supplementary Table S6. List of somatic mutations detected by targeted sequencing in cfDNA in Hispanic study participants with APRI≥1. Ref_allele: base in the reference genome; Alt_allele: base altered in the sample; Chr.: chromosome; Exonic func: Function of exonic mutation; SNV: single nucleotide variants; Aaannotation: amino acid change; Tumor_f: allele frequency; ns-SNV: nonsynonymous SNV; f-deletion: frameshift deletion; nf-deletion: nonframeshift deletion; nf-insertion: nonframeshift insertion; N: number of subjects with mutated gene.

Gene (N)	Start_ position	Ref_ allele	Alt_ allele	Chr.	Exonic func	Aaannotation	Tumor_f
KMT2C (3)	151859521	T	C	7	ns-SNV	p.K3714R	0.47
	151932990	C	T	7	ns-SNV	p.R894Q	0.05
	151970859	C	A	7	ns-SNV	p.G315C	0.07
ATM (2)	108188136	G	A	11	ns-SNV	p.V2079I	0.43 ; 0.50
	108190770	G	C	11	ns-SNV	p.S2146T	0.49 ; 0.48
BRCA1 (2)	41243509	T	C	17	ns-SNV	p.R1347G	0.49 ; 0.46
TPRXL (2)	14106332	G	C	3	ns-SNV	p.S219T	0.15 ; 0.46
MYD88 (2)	38181420	G	A	3	ns-SNV	p.A145T	0.51 ; 0.5
HLA-F (2)	29691687	A	G	6	ns-SNV	p.Y106C	0.53 ; 0.53
TET2 (2)	106157616	0	-	4	f-deletion	p.H860fs	0.16 ; 0.07
FOXD4 (2)	117846	A	T	9	ns-SNV	p.S92T	0.42
	117873	C	T	9	ns-SNV	p.G83S	0.2
MUC21 (2)	30954324	G	C	6	ns-SNV	p.E124D	0.11
	30954845	A	G	6	ns-SNV	p.N298S	0.17
NOTCH3 (2)	15272337	0	-	19	f-deletion	p.P2034fs	0.06
	15311605	0	-	19	f-deletion	p.A38fs	0.05
APC (2)	112175253	0	-	5	f-deletion	p.S1321fs	0.43
	112175430	C	G	5	ns-SNV	p.T1380S	0.04
ASTN1 (1)	176903324	G	A	1	ns-SNV	p.R879W	0.11
MMEL1 (1)	2560821	G	C	1	ns-SNV	p.L35V	0.07
C1orf95 (1)	226736652	C	T	1	ns-SNV	p.A16V	0.18
WHSC1L1 (1)	38162945	G	A	8	ns-SNV	p.S754L	0.5
NOTCH2NL (1)	145273354	T	C	1	ns-SNV	p.C70R	0.16
RANBP1 (1)	20114511	C	T	22	ns-SNV	p.A181V	0.52
ROS1 (1)	117686261	C	T	6	ns-SNV	p.G1027D	0.5
TECPR1 (1)	97870222	C	T	7	ns-SNV	p.G292R	0.54
JAK2 (1)	5126343	G	A	9	ns-SNV	p.R1063H	0.48
ERBB3 (1)	56495562	C	G	12	ns-SNV	p.T1251S	0.51
HSP90AA1 (1)	102552202	G	A	14	ns-SNV	p.A263V	0.42
MAP2K1 (1)	66781553	C	T	15	ns-SNV	p.P321S	0.49
RBFOX1 (1)	6367045	G	A	16	ns-SNV	p.R18Q	0.48
JAK3 (1)	17943694	G	A	19	ns-SNV	p.R799C	0.52
BMP2K (1)	79792085	G	C	4	ns-SNV	p.Q460H	0.07
KMT2D (1)	49434075	C	T	12	ns-SNV	p.G2493E	0.57
DICER1 (1)	95570180	C	T	14	ns-SNV	p.A1185T	0.42
MUC6 (1)	1017768	G	A	11	ns-SNV	p.T1678I	0.18

RUNX3 (1)	25255983	C	T	1	ns-SNV	p.R140K	0.39
ERBB2 (1)	37879585	A	G	17	ns-SNV	p.I654V	0.46
TGFBR1 (1)	101911500	G	T	9	ns-SNV	p.W479C	0.49
AMER1 (1)	63410516	G	A	X	ns-SNV	p.P884L	0.48
PDGFRA (1)	55131164	C	A	4	ns-SNV	p.A236D	0.13
SPEN (1)	16257476	G	T	1	ns-SNV	p.V1581F	0.46
IDH1 (1)	209108311	T	C	2	ns-SNV	p.M180V	0.54
PPP1R3A (1)	113518529	T	C	7	ns-SNV	p.K873R	0.49
GNAS (1)	57428423	G	C	20	ns-SNV	p.G35R	0.16
	57429413	G	A	20	ns-SNV	p.A365T	0.43
TP53 (1)	7579419	0	-	17	f-deletion	p.S105fs	0.41
ALK (1)	29416378	0	-	2	nf-deletion	p.1525_1525del	0.44
ELMSAN1 (1)	74205941	-	TGCTG CTGT	14	nf-insertion	p.Q257delinsQ QQQ	0.34
NTRK1 (1)	156849112	0	-	1	f-deletion	p.D668fs	0.06
CDC27 (1)	45229185	C	G	17	ns-SNV	p.G359R	0.04
